# The Effect of a G:T Mispair on the Dynamics of DNA

**DOI:** 10.1371/journal.pone.0053305

**Published:** 2013-01-15

**Authors:** Petra Imhof, Mai Zahran

**Affiliations:** Interdisciplinary Center for Scientific Computing, Computational Molecular Biophysics, University of Heidelberg, Heidelberg, Germany; German Research School for Simulation Science, Germany

## Abstract

Distortions in the DNA sequence such as damages or mispairs are specifically recognized and processed by DNA repair enzymes. A particular challenge for the enzymatic specificity is the recognition of a wrongly-placed native nucleotide such as thymine in T:G mispairs. An important step of substrate binding which is observed in many repair proteins is the flipping of the target base out of the DNA helix into the enzyme’s active site. In this work we investigate how much the intrinsic dynamics of mispaired DNA is changed compared to canonical DNA. Our molecular dynamics simulations of DNA with and without T:G mispairs show significant differences in the conformation of paired and mispaired DNA. The wobble pair T:G shows local distortions such as twist, shear and stretch which deviate from canonical B form values. Moreover, the T:G mispair is found to be kinetically less stable, exhibiting two states with respect to base opening: a closed state comparable to the canonical base pairs, and a more open state, indicating a proneness for base flip. In addition, we observe that the thymine base in a T:G mispair is significantly more probable to be flipped than thymine in a T:A pair or cytosine in a C:G pair. Such local deformations and in particular the existence of a second, more-open state can be speculated to help the target-site recognition by repair enzymes.

## Introduction

Deamination of cytosine or methyl-cytosine is a DNA damage resulting in mutation to uracil or thymine, respectively, thus leading to G:U or T:G mismatches. Glycosylases such as the human thymine DNA glycosylase (TDG) or uracil DNA glycosylase (UDG) recognize T:G or G:U mismatches and remove specifically the mispaired T or U, respectively. Detection of a T:G mispair means recognition of one of the four nucleotides which compose the DNA but is positioned at a wrong site, hence resulting in sequence mismatches.

From the many structures of glycosylases complexed to damaged DNA [1], [2] it is known that damaged, mispaired or wrong bases are flipped out of the helical DNA duplex into the enzyme’s active site. This base flip has been argued [3], [4] to facilitate access to proton acceptor groups of the scissile base, i.e. the base which will be removed by the repair enzyme. In the debate how glycosylase enzymes recognize a damaged or mispaired base two mechanisms are discussed. One is a “passive” mechanism in which the enzyme detects extra-helically exposed, already (at least partially) flipped-out bases. This mechanism implies that base pair opening up to several degrees of flipping is more likely for damaged/mispaired bases than for intact canonical ones. The alternative mechanism involves flipping of the base while the enzyme travels along the DNA, relying on the enzyme specifically enhancing the flip-out of its target base [4], [5].

Solution NMR studies have shown that the T:G mispair introduces only local perturbations to the DNA B form not extending beyond the neighbouring base pairs [6]. Besides small deviations in the backbone torsion angles the authors report an asymmetry of the 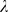-angles between the glycosydic bonds and the base pair vector (C1’-C1’) for T:G mismatches as opposed to the rather symmetric 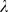 angles observed in canonical base pairs [6].

An experimental probe for base opening and re-closing of hydrogen bonded base pairs is the exchange of imino protons of guanine, uracil or thymine which can be measured by NMR [7]–[9]. The base-opening rate can be calculated from the imino proton exchange rate assuming that both rates are equal if the exchange itself is fast (which can be achieved by the use of proton accepting catalysts). However, imino proton exchange rates cannot be directly used as a measure for base flipping since solvent accessibility of the imino protons can be achieved already at low flip (opening) angles [10]–[12]. Moreover, as imino-proton accessibility can be achieved by flipping of either of the two bases in a pair, the kinetics of a single base flipping completely out of the DNA double helix - towards the conformation which has been observed in complexes with DNA repair proteins - can thus not be directly extracted from the experiments.

Molecular simulations have proven to be a powerful tool for obtaining information on structure and dynamics at the atomic level which is not directly accessible to experiments, and have been used successfully to analyse conformational changes in proteins and DNA [13]–[27].

Sequence dependent dynamics of uncomplexed DNA with different types of damage, lesion or mispairs, has been investigated by numerous molecular dynamics studies [15], [28]–[37]. Simulations of base flip have been successfully conducted on free DNA [28]–[40] and in complex with different DNA repair enzymes [21], [22], [41], [42].

Crystallisation experiments on human Thymine DNA glycosylase solved the structure of a protein-DNA complex with a flipped-out product analogue [43]. Kinetic experiments showed the importance of conserved residues for base flipping [44]. Recent work by the same group revealed the structure of a DNA with a substrate-analogue (U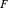) [45] complexed to wild-type and mutant protein. The authors moreover report MD simulations of the protein-DNA complex in the flipped-out form, showing that two conserved residues destabilise the completely-flipped form of target dT as opposed to substrate dU. They conclude this incomplete flip to reduce the accessibility of the dT, which could minimise aberrant T removal from A:T pairs. This is consistent with earlier biochemical work by the same authors [46] in which the reversible nucleotide flipping was found to be much more rapid for G:T than for G:U substrates.

Since flipping of a DNA base occurs on time scales much larger than accessible by direct MD simulations various flavours of enhanced molecular dynamics have been used which all force the base flip to occur by applying an external potential. The main difference is the definition of the reaction coordinate, i.e. the geometric parameter (e.g. internal coordinate) being restrained. For a detailed review of different methods see [29], [40]. Comparisons of the popular force fields has been reported in [33]. The two popular force fields CHARMM [47] and AMBER parm99 [48] show similarly good agreement with experimental imino proton exchange data. However, the detailed atomic picture as well as the calculated PMFs vary between the different force fields. In a more recent study [35] long simulations of long DNA duplexes with the CHARMM force field and with the improved Amber force field parmbsc0 [49] have been compared. The authors conclude a “very remarkable similarity between parmbsc0 and CHARMM27 estimates” for helical stiffness. Hydrogen bonds between Watson-Crick pairs C:G and T:A are found to be less strong with CHARMM27 compared to parmbsc0. Transient loss of hydrogen bonds is observed to be common using either of the two force fields. In conclusion both force fields are of similar quality to obtain a “consensus picture of the basic structural dynamics characteristics of B-DNA” [35].

The opening of T:G pairs in DNA (and G:U in RNA) has been studied recently, by performing a combination of imino proton exchange measurements and molecular dynamics simulations [12]. The authors defined opening by a linear combination of the flip angles of the two bases G and T. The energetically most favourable opening pathway was found to be a coupled rotation of both bases, opening through the major groove. Moreover, the authors conclude that the common two-state model for base pair opening can be applied since imino protons of the closed pair are found to be not accessible for the solvent. However, proton exchange was reported to take place with only 10–40% accessibility [12].

In this work, we apply molecular simulations to explore how much the intrinsic dynamics of the mispaired DNA, compared to intact, well-paired DNA, contribute to mispair recognition by repair proteins. We have performed molecular dynamics simulation of short sequences of unbound DNA in water in order to investigate the dynamics of the paired DNA and of DNA carrying one T:G mispair instead of a G:C pair or an A:T pair. We have analysed the conformational difference of the DNA at the T:G mispair compared to Watson-Crick pairs C:G and T:A. Furthermore, we have computed the free energy for the non-enzymatic flip process in water in order to investigate whether a thymine from a T:G mispair can be flipped out of the DNA double helix more easily than flipping cytosine from C:G or thymine from T:A.

## Methods

### System Setup

Three setups of DNA oligonucleotides of 17 base pairs length were prepared in standard B-DNA form, d(GCTCTGTACGTGAGCAG), the site of interest is underlined. This is the part of the DNA sequence observed in the crystal structure of the hTDG-DNA complex (2RBA [43]) where the protein is bound to. The site which is abasic in the hTDG-DNA complex has been modelled with either cytosine (C:G) or thymine (T:G), both flipped in. As another reference, a third setup with an T:A pair at the C/T:G site was prepared. These initial models were build and minimised with CHARMM [47]. The CHARMM 27 Force field [50] was used throughout.

The 17-bps oligomers have a length of ∼60 Å and a width of ∼20 Å. The systems were solvated with explicit water, using the TIP3P model [51], extending to at least 10 Å beyond the DNA in each direction in a cubic box (x = y = z = 90 Å). The cubic shape ensures that even after rotation there would be enough distance between two adjacent images. 36 Na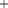 counter-ions were added to neutralize the system and an excess of Na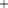 and Cl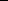 ions to obtain a physiological concentration of 150 mM NaCl. The addition of the ions was carried out by random substitution of water oxygen atoms.

Simulations were performed using periodic boundary conditions and the long-range electrostatic interactions were treated using the Particle Mesh Ewald method [52] on a 92×92×92 charge grid, with a non-bonded cutoff of 12 Å. The short range electrostatics and van der Waals interactions were truncated at 12 Å using a switch function starting at 10 Å.

The solvated structures were minimized using 5000 steps of steepest descent, followed by minimization with the conjugate gradient algorithm, with solute atoms harmonically restrained until an energy gradient of 0.01 kcal/(mol Å) was reached. The system was then gradually heated for 30 ps to 300 K with 1 K temperature steps with harmonic restraints on the solute atoms.

The systems were equilibrated in three different stages with the numbers of particles, pressure (1 bar) and temperature kept constant (NPT ensemble) during 75 ps. In the first 25 ps velocities were rescaled every 0.1 ps and in the second 25 ps Langevin dynamics were used to maintain constant temperature. Pressure control was introduced in the third 25 ps and in the production run using the Nosé-Hoover Langevin piston with a decay period of 500 fs [53]. The harmonic restraints were gradually lifted (to 0.5, 0.25 and 0.05 kcal/(mol Å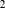)) in the three equilibration stages.

### Unbiased MD Simulations

After equilibration, unbiased NPT production runs were performed for 60 ns. The integration time step was 2 fs and coordinates were saved with a sampling interval of 2 ps. All covalent bonds lengths involving hydrogen atoms were fixed using SHAKE algorithm [54].

Several independent MD simulations were carried out by assigning different initial distributions of starting velocities to the minimized systems: three runs for the two setups of paired DNA (C:G and A:T), and five for the mispaired T:G model (cf. Table 1).

**Table 1:** List of MD simulations.

	unbiased	ABF
C:G	3*60 ns	3*30ns
T:G	5*60 ns	5*32ns
T:A	3*60 ns	3*30ns

### Biased (ABF) MD Simulations

**Figure 1:**
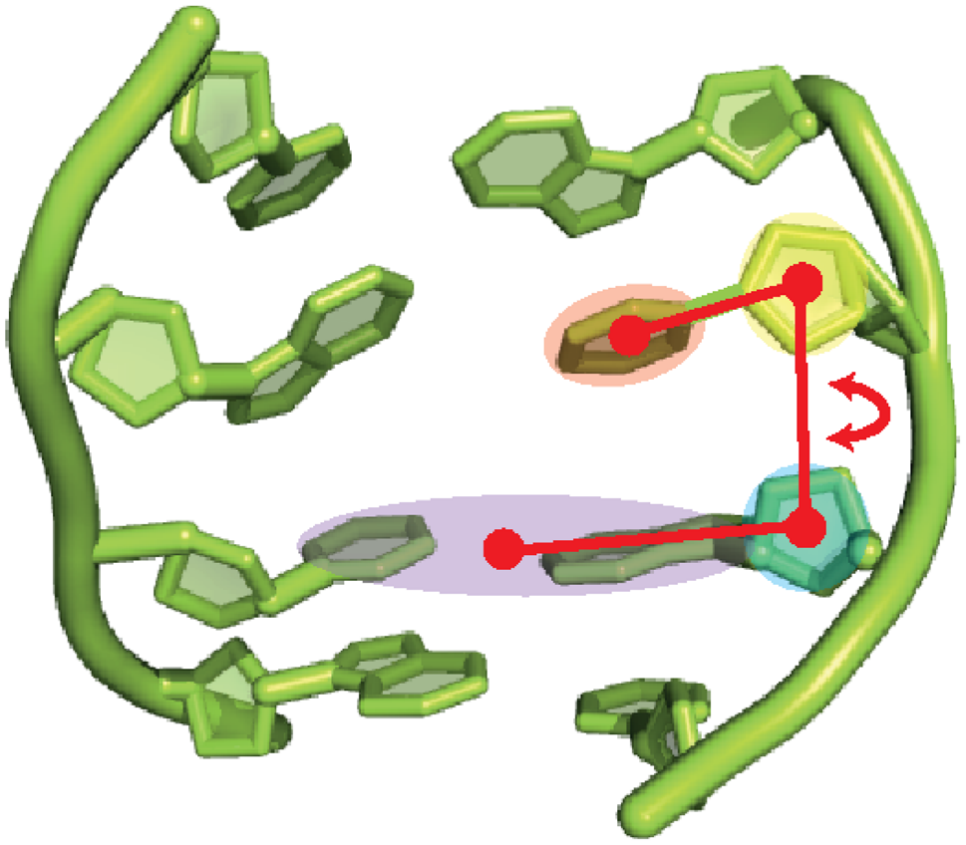
Definition of the reaction coordinate for the base flip simulations: the flip angle is a pseudo-dihedral between the centres of mass of the flipping base (red shade), the sugar moiety of the same nucleotide (green shade), the sugar moiety of the next nucleotide (yellow shade), and the base of the next nucleotide plus the complementary base in the other DNA strand (blue shade).

For the simulation of the base flip we applied the Adaptive Biasing Force (ABF) method [55]–[57]. In ABF the reaction coordinate is discretized into small bins. Sampling is carried out along the reaction coordinate in a continuous fashion. In each bin samples of the instantaneous force acting along the reaction coordinate are accrued up to a certain threshold. If this threshold is reached the adaptive biasing force is applied so as to “drive” the system into the next bin. The reaction coordinate for the base flip has been defined as a pseudo-dihedral angle between the flipping base, the sugar moiety of the same nucleotide, the sugar of the next nucleotide, and the base of the next nucleotide plus the base and sugar of the opposing nucleotide downstream (see Figure 1). This definition of the flipping coordinate is similar to the one proposed and applied in [31], [33], [38]. The potential of mean force (free energy profile) was obtained by discretising the reaction coordinate between 10° and 180° into windows of 2° width, and in each window 2000 samples were collected before the bias was applied. For C:G and T:A we carried out three ABF simulations each, and for T:G five individual ABF simulations were started with different initial velocities. The biased simulations were run for 24 ns each (cf. Table 1). All molecular dynamics simulations were performed with the NAMD [58] program.

### Analysis

To calculate potentials of mean force, angles were binned by 2 degree and translation parameters were binned by 0.2 Å. The free energy difference, 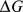, to the reference state was evaluated according to
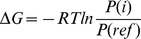
where 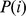 is the probability of finding the system in state 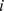 and 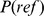 is the probability of finding the system in the reference state. Probabilities have been calculated from the number of occurrences within a bin. The bin with the highest occurrences has been chosen as the reference state. The free energy has been evaluated in the 99% confidence interval.

For all analyses (unbiased and ABF simulations), properties were evaluated for each run individually. Then the averages and standard errors over the respective individual runs were calculated.

In the analyses of the unbiased MD simulations, the first 10 ns of each trajectory were not included. The Root mean Square Deviation (RMSD) as a function of time, plotted in Figure S1 in the supplementary material suggests this simulation time to be sufficient. The convergence of these simulations was furthermore evaluated by comparison of the properties computed from different simulation lengths, i.e. 40, 50 and 60 ns (shown as Figures S5–S17 in the supplementary material). Convergence of the ABF simulations has been evaluated in a similar manner by comparing the free energy profiles obtained after 20 ns –32 ns simulation time (cf. Figure S19 in the supplementary material).

The conformations of the paired and mispaired DNA were characterized by calculating twelve helical parameters, six for base steps (the three rotational parameters: roll, tilt and twist, and the three translational parameters: slide, rise and shift) and another six for base pairs (buckle, propeller, opening, stagger, shear, stretch) that define the local DNA geometry. In addition, we have computed the lambda angles which define the angles between the glycosidic bonds (N1/N9–C1’) and the base-base vector (C1’–C1’).

Hydrogen-bond occupancies were calculated as the ratio of the time when the hydrogen bond is formed to the total time of the trajectory. Two atoms are considered here to form a hydrogen bond if the acceptor-donor distance is 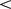
3.0 Å and the acceptor-hydrogen-donor angle is 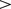
135.

Solvent accessible surface areas have been computed by placing a probe sphere of radius r
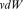
+1.4 Å in contact with the atomic van der Waals sphere, both centred at the atom. The parts of the surface spheres where the centre of the spherical probe can be placed without penetrating other atoms add up to the solvent accessible surface area [59].

The Molecular Dynamics simulations have been carried out using the program NAMD2.7 and applying the CHARMM27 force field. All simulations have been performed on the local group cluster, the Heidelberg Linux Custer (HELICS) and the North-German Supercomputing Alliance (HLRN).

All molecular images were generated with the molecular visualization program VMD [60] and with the molecular graphics program Pymol [61]. Structural analysis was performed using standard programs; Curves5.3, gromacs-4.5.5 [62]–[64] tools and our own scripts.

## Results

### DNA Conformation

**Figure 2:**
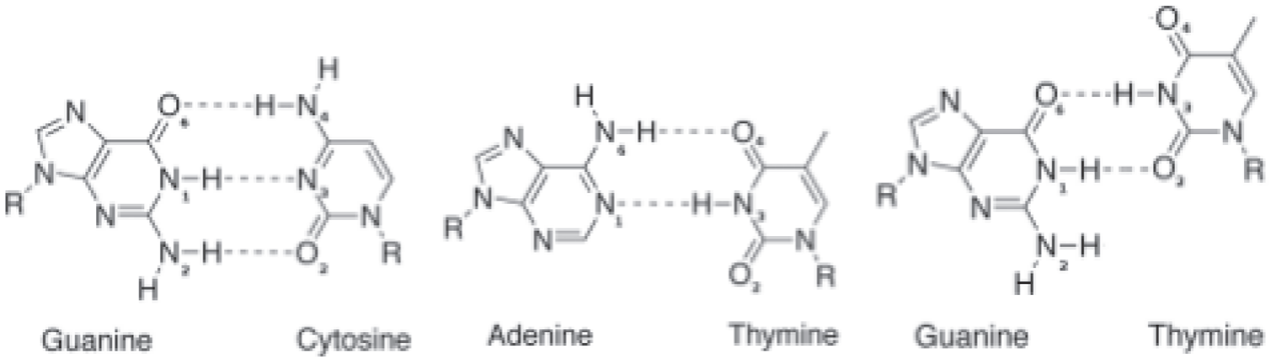
Schematic drawing of Watson-Crick pairs C:G (left) and T:A (middle), and the mispair T:G (right).

**Figure 3:**
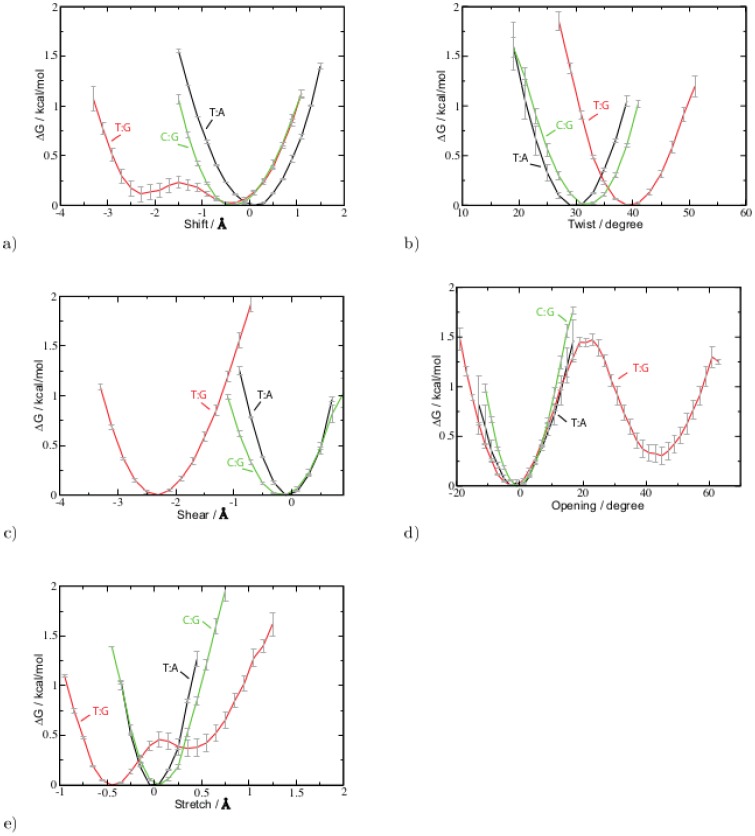
Free energy profiles of local DNA parameters of the T:G mispair (red) and C:G (green) and T:A (black) pair, respectively, obtained from unbiased MD simulations. Only those parameters with significant differences are shown a) shift b) twist c) shear d) opening e) stretch. For the free energy profiles of the other parameters see Figures S2 and S3 in the supplementary material.

We have examined the conformation of the DNA double helix carrying the mispair and compared it to the intact DNA, analysing the local conformation at the mispair T:G, the pair C:G and for comparison at a T:A pair (see Figure 2 for a schematic drawing of the three base pairs). Figure 3, and Figures S1 and S2 show the free energy profiles for the local helical parameters representing the local DNA conformation at the T:G, C:G and T:A base pair, respectively. Among the helical parameters characterizing the base step only the twist angle and the shift translation exhibit significant differences between the C:G or T:A pairs and the mispaired T:G. The twist angle has a free energy minimum at 32
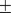
1° and 30
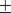
1° for the two Watson-Crick pairs, C:G or T:A, respectively, whereas for the T:G wobble pair a higher twist angle (39
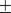
1°) is more probable. In case of the shift translation, the T:G mispair shows two free energy minima. The first one is located at around −0.5
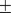
0.2 Å, at about the same position as the free energy minimum of the C:G shift (−0.4
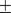
0.1 Å) and close to the 0.2
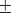
0.1 Å shift of the T:A pair. The second free energy minimum of the T:G mispair, which is only marginally higher in energy than the first one, is observed at −2.3
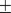
0.4 Å.

The free energy profiles of the base-pair parameters computed from the unbiased simulations of the three different DNA setups again show high similarities between the two Watson-Crick pairs C:G and T:A (see Figures 3, S2 and S3). The most pronounced differences between Watson-Crick pairs and the wobble pair can be observed for the shear and stretch translations, and for the opening angle. T:G shows a free energy minimum for shear at −2.3
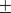
0.1 Å and the most probable stretch translation at −0.5
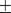
0.05 Å, which deviate from the value of the Watson-Crick pairs by −2 Å and −0.5 Å, respectively. Moreover, in the T:G case, a second free energy minimum for the base-pair stretch (0.4
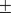
0.1 Å) can be observed, albeit with higher statistical errors. The free energy profile of the base pair opening angle also shows that the T:G wobble pair has (at least) two states. The most likely state has an opening angle similar to that of the Watson-Crick pairs (−1.0
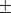
0.5 Å). However, a second, slightly less probable state which is separated by only 1.5 kcal barrier, is observed at an opening angle of 45
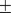
1° (Figure 3). The distortions on the DNA carrying the T:G mispair are very local as can be seen from the comparison of the local parameters of the flanking bases and base pairs (supplementary material, Figures S5, S6, S7, S8, S9, S10, S11, S12, S13, S14, S15, S16). The only parameter of neighbour which is affected is the shift of the base step preceding the mispaired T. This is due to the definition of the shift, i.e. the displacement along the x-axis with respect to the neighbouring base step. The displacement of base number 10 (T) leads to a shift with respect to base number 11 and base number 9.

### Hydrogen Bonds

**Figure 4:**
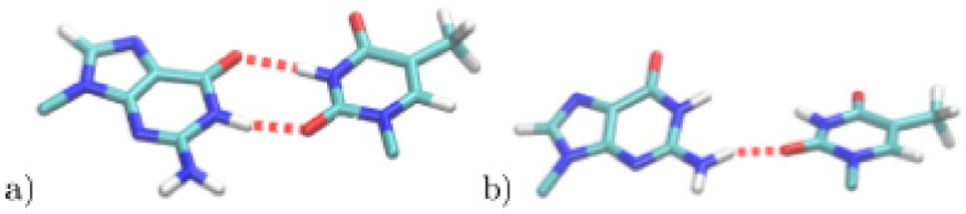
Snapshots of the unbiased simulation of T:G. a) Two-hydrogen bonds conformation, b) one-hydrogen bond conformation.

Table 2 lists the occupancies of hydrogen bonds of the target base with the base on the complementary DNA strand or with solvent water, respectively. As anticipated the hydrogen bonds in the Watson-Crick pairs C:G and T:A are very stable as manifested by the hydrogen bond occupancies of 78
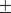
1% and 96
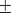
1%. Only the C-N4–G-O6 bond in the C:G pair is more dynamic and is formed 68
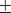
5% of the simulation time. The T:G mispair shows one very stable hydrogen bond between G-N1 and T-O2 which is formed 72
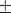
3% of the simulation time. Another hydrogen bond to T-O2 is formed by G-N2 with an occupancy of 34
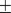
6%, suggesting that the T-O2 fluctuates between the two hydrogen bonded states (see Figure 4).

**Table 2:** Occupancies of hydrogen bonds between DNA base pairs C:G, T:A, and mispair T:G, computed from the simulation of paired and mispaired DNA.

paired (C:G)			mispaired (T:G)			paired (T:A)		
Donor	Acceptor	Occ./%	Donor	Acceptor	Occ./%	Donor	Acceptor	Occ./%
G-N2	C-O2	90 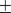 2	G-N2	T-O2	34 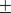 6	A-N6	T-O4	78 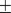 1
G-N1	C-N3	91 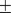 4	G-N1	T-O2	72 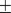 3			
C-N4	G-O6	68 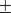 5	T-N3	G-O6	50 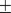 6	T-N3	A-N1	96 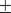 1
								
water	C-O2	68 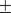 29	water	T-O2	17 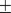 3	water	T-O2	65 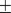 3
C-N4	water	56 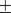 4	water	T-O4	71 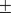 3	water	T-O4	57 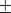 9
			T-N3	water	35 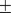 7			

Hydrogen bonds of C and T with bulk water are listed, too. G and A are the corresponding bases on the complementary strand in the C:G and T:A pair, respectively. For atom labels see Figure 2.

T-N3 is observed to form hydrogen bonds to G-O6 about half of the simulation time(50
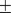
6% occupancy). The imino proton (N3-H) of thymine also forms hydrogen bonds to solvent water, for about the same percentage of the simulation time as the T-O2–G-N2 hydrogen bond is occupied (35
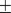
7% and 34
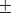
6%, respectively).

The analysis of the hydrogen bonds with water shows that all oxygen atoms involved in hydrogen bonds within the base pair (C-O2, T-O2 and T-O4) accept additional hydrogen bonds from solvent water. The amino group nitrogen atom C-N4 acts as hydrogen bond donor to solvent water, too. A significant difference, however, is the observation of hydrogen bonds between T-N3 and water in the mispaired Thymine only.

### Base Flip

**Figure 5:**
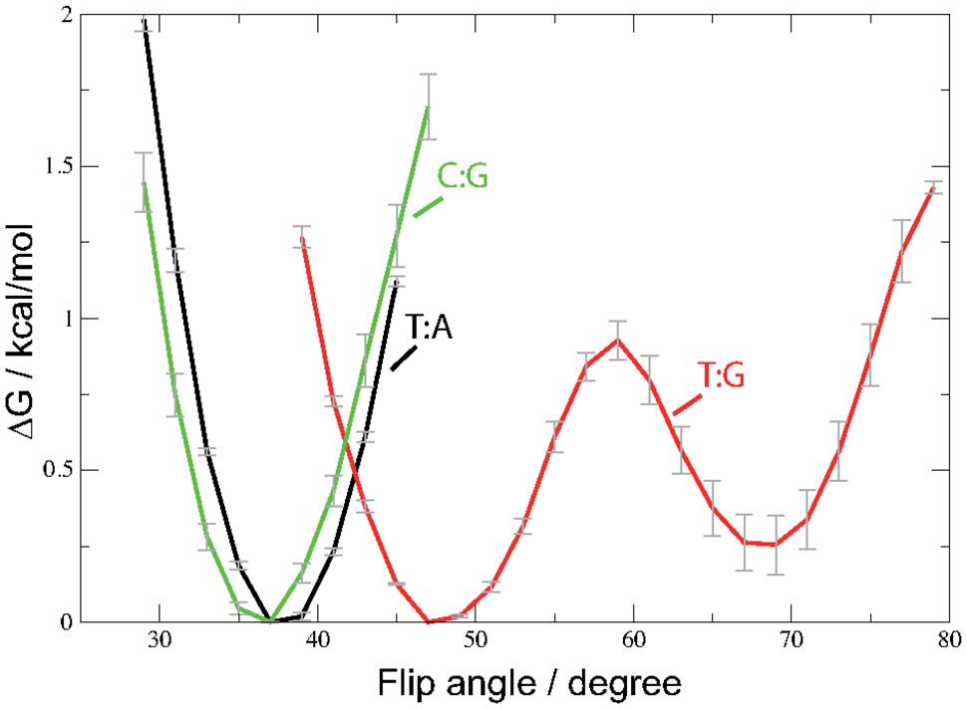
Free energy profiles of the pseudo-dihedral flip angle evaluated from the unbiased MD simulations of the T:G mispair (red) and C:G (green) and T:A (black) pair, respectively.

Figure 5 shows the free energy profile of the pseudo dihedral flip angle for the three setups T:A, T:G, and C:G computed from the unbiased MD simulation. Whereas the Watson-Crick base pairs C:G and T:A exhibit a rather narrow free energy minimum around 37
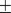
1° and 38
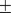
1°, two free energy minima can be observed in the free energy profile of the mispaired T:G DNA. The most probable flip angle is at 47
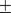
1° and a second free energy minimum, about 0.3 kcal/mol higher in energy is located at 68
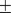
1°. The rather low barrier between the two free energy minima allows for frequent transitions to be observed (cf. Figure S18).

**Figure 6:**
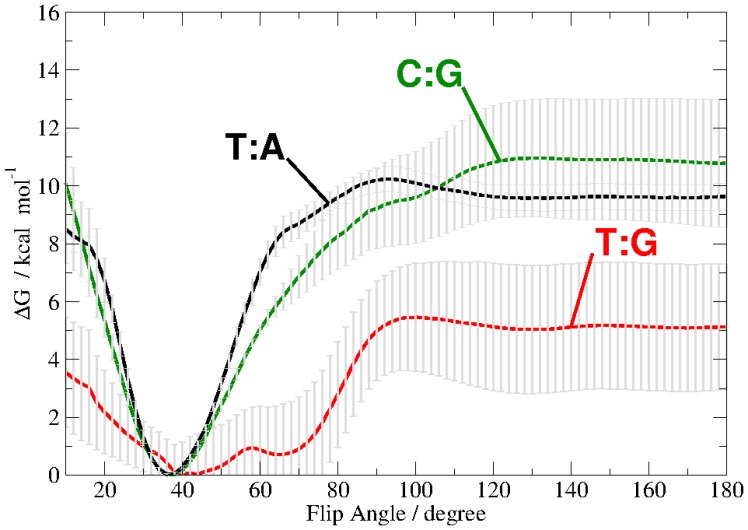
Free Energy profile of the base flip for thymine of a T:G mispair (red), cytosine of a C:G pair (green) or thymine of a T:A pair (black). The pseudo dihedral coordinate is illustrated in Figure 1.

By applying the technique adaptive biasing force, we have computed the free energy for the rotation (flip) of a single base out of the DNA double helix up to 180 degree flip angle. A complete rotation of the DNA base, including passage of the minor groove turned out to require too high forces resulting in deformation of the DNA. Therefore, we have computed the potential of mean force only for a flip through the major groove. The free energy profile is plotted in Figure 6.

The positions of the free energy minima are the same as those observed in the unbiased simulation for the two Watson-Crick base pairs. The thymine base of the mispaired T:G, however, exhibits two free energy minima at 42
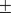
6° and at 67
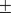
2°, separated by a barrier of ∼1 kcal/mol. These free energy profiles are comparable to those obtained from the unbiased simulation (Figure 5).

As anticipated, the flip of a cytosine base from a C:G pair requires significantly more energy (on average 11
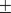
2 kcal/mol) than the flip of a mispaired thymine (5.5
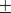
1.8 kcal/mol kcal/mol). The flip of a thymine base from a T:A pair has only slightly lower free energy barrier but a significantly narrower error range (10.4
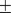
0.5 kcal/mol) than the cytosine flip of the C:G pair.

### Water Accessibility

**Figure 7:**
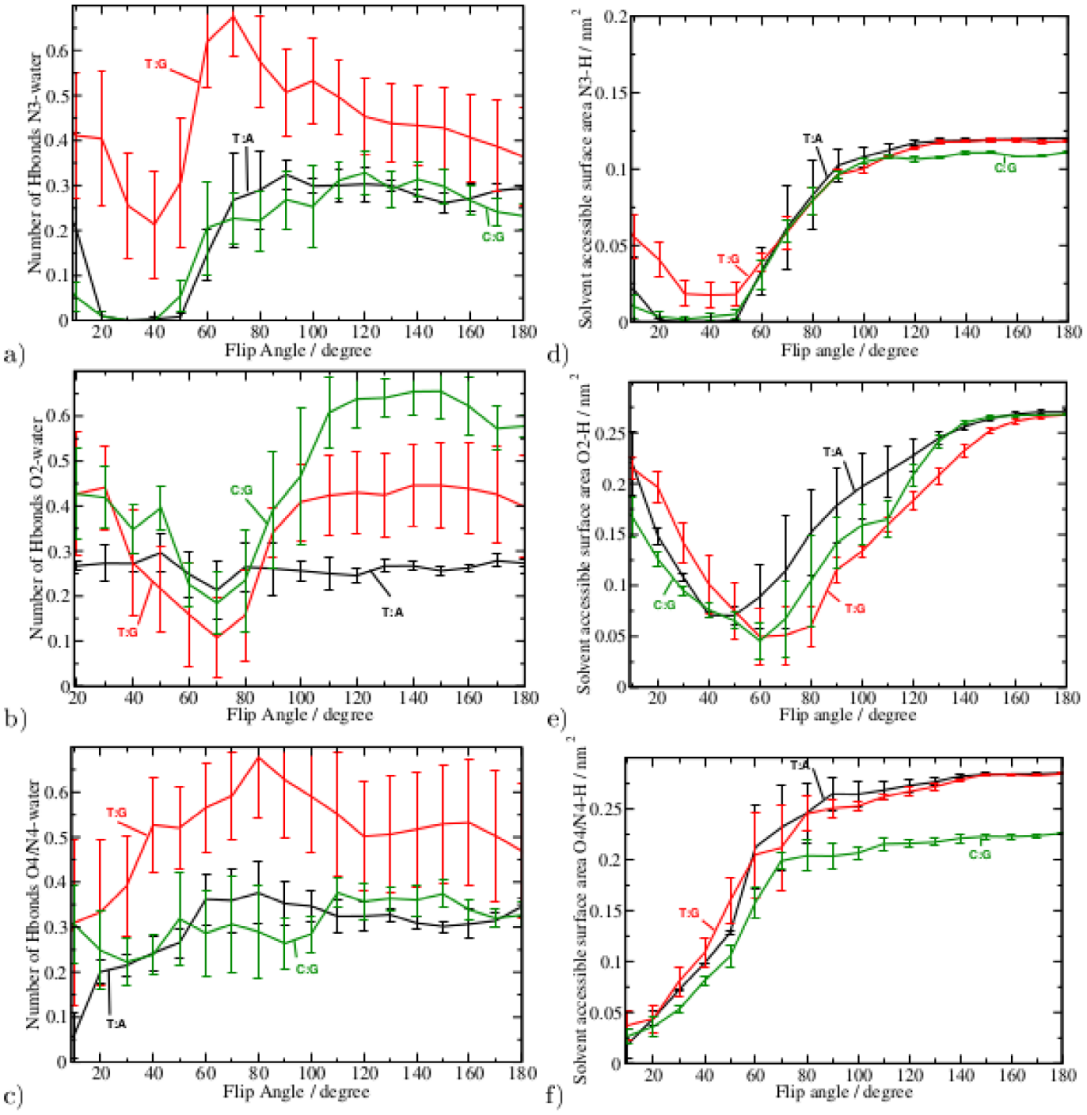
Left: Average number of Hydrogen bonds of solvent water with base atoms a) N3-H b) O2 and c) O4 or N4H2 in case of thymine or cytosine, respectively, as a function of the base flip angle. Right: Solvent accessible surface area of base atoms d) N3-H e) O2 and f) O4 or N4H2 in case of thymine or cytosine, respectively, as a function of the base flip angle. Data from the simulation of the C:G and T:A pairs are plotted in black and green, respectively, data for the T:G mispair is shown in red. For atom labels see Figure 2.

In order to analyse how the base flip affects the otherwise shielded intra base-pair hydrogen bonds we have computed the water accessibility of the hydrogen bond formed with atoms of cytosine and thymine (N3, O2, N4, and O4, respectively) as a function of the base flip angle. The respective curves are plotted in Figure 7, left. The additionally computed water accessible surface area of those atoms is shown in Figure 7, right. Both, hydrogen bonds and solvent accessible surface area show a similar dependency on the base flip angle, except for the O4/N4H2-atom. For the N3-atom both curves show minima at about the same flip angle (35
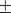
° for C:G and T:A, and 40
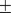
5° for T:G, respectively) as the free energy profile. However, the minima of hydrogen bonds with the N3 atom and solvent accessible surface area of the T:G mispair are significantly narrower than the free energy minimum of the flip angle. T:G exhibits an average number of 0.3 hydrogen bonds between the N3-atom and solvent water even at the free energy minimum flip angle (40
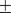
5°) which is in agreement with the 35
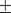
7% hydrogen bonds occupancy observed in the unbiased simulations (cf. Table 2).

In the two cases where O2 is hydrogen-bond to guanine (G:C and T:G) the water-O2 interaction (either in terms of numbers of hydrogen bonds or as solvent accessible surface area) is minimal at a flip angle of 70
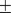
10° and 60
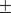
10°, respectively. The O2-atom of thymine is not involved in a Watson-Crick interaction when paired to adenine, and is then able to form hydrogen bonds with solvent water in a flipped-in state. Hence, the number of hydrogen bonds does not change significantly with respect to the flip angle. Its solvent accessibility, however, is minimal at the flipped-in state (45
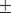
10° flip angle) and increases with flip angle similar to the free energy for base flipping.

Atoms O4 and N4 show an increase of numbers of hydrogen bonds and an increase of the solvent accessible surface area at a flip angle of up to 60–70°. The exceptions are the number of hydrogen bonds formed between the mispaired thymine O4 and solvent water, and between N4H2 with water via the second hydrogen atom of, which both are already present in the flipped-in state (see also Table 2).

Figure S21 in the supplementary material shows snapshots of the base flip trajectories. In the flipped-in state the thymine/cytosine base forms two/three hydrogen bonds to its complementary base. At a flip angle of 60–70° only the O2-atom is buried in the DNA double helix and forms a hydrogen bond to the opposite base on the complementary strand. At 180° flip angle, the base is completely flipped out into the solvent, whereas neighbouring bases are properly paired.

## Discussion

The analysis of the DNA helical parameters clearly shows a distortion in the DNA containing the T:G mispair compared to canonical DNA. In particular the base pair parameters shear and stretch show a significant deviation from the values observed for the Watson-Crick pairs. A higher twist angle is another indicator for the mispaired base. The wobbling of the T:G mispair is manifested in several respects. The hydrogen-bond occupancies indicate that the O2-atom of T alternates between being hydrogen bonded to the N1 (imino) or N2 (amino) nitrogen atom of guanine suggesting that T:G has two metastable states. The first state has two, the latter, less probable state has one hydrogen bond between the two bases. This two-state behaviour is also represented in the two free energy minima of shift and shear. The base pair opening angle exhibits a second free energy minimum, too, which is located at a significantly higher angle than the first minimum or the free energy minima of the Watson-Crick pairs.

The molecular dynamics simulations of the T:G mispair show significant local distortions comparable to those found by NMR experiments [6]. We observe 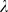-angles similar to those reported in [6] for all three base pairs (cf. Figure S4). However, in our simulations the mispair has a second state with a much higher 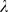-angle. Moreover, the wobble pair is kinetically unstable and fluctuates between two states, one of which is closer to canonical B-form conformation than the other. The more distorted state is less probable and as such can be regarded as only transiently occupied. This second state has not been observed in the structures of T:G containing DNA, modelled from solution NMR data. This discrepancy can be due to overestimation of the open state by the force field used in the simulations and/or underestimation due to the restraints applied in the structure modelling based on the NMR spectra. The occasional (partial) openings of the base pair as observed in our simulations may be sufficient to be detected by the searching glycosylase and induce further inspection by the enzyme such as transitions from an “interrogating complex” to an “excision complex” [5] including flipping the base.

The free energy profile computed for the base flipping of thymine or cytosine out of T:G, T:A or C:G pairs, respectively, shows that the mispaired thymine is the most probable to reach an extra-helical conformation. The energy required for the single base flip (5 kcal/mol) is about the same as computed previously for the combined rotation of both, G and T, opening through the major groove [12]. The free energy cost for flipping a single T out of a T:A pair (10 kcal/mol) and C out of a C:G pair (11 kcal/mol) are similar to each other but significantly higher than that computed for mispaired thymine. The error range for the cytosine flip in the higher flip angle regime, however, is significantly larger than for thymine from T:A pairs. This is due to higher energy pathways having been sampled and also indicates a larger conformational heterogeneity of the flipped-out cytosine. Note that the free energy profile shown in Fig. 6 is computed from averaging over several independently calculated free energy profiles. In case of the T:G mispair these individual runs differ from each other in terms of the exact location of the two free energy minima and the barrier between them (cf. Figure S20). This variance indicates that another conformational change, taking place on longer time scales, has not been fully averaged out in the individual runs. However, the similarity between the profiles of the flip angle obtained for the unbiased and the averaged ABF simulations suggests that by averaging over several individual runs the insufficient sampling of the individual runs could be compensated to some extent. Among the many possible “slow degrees of freedom” a flip and re-stacking of the complementary base are the most probable candidates. We have observed such transitions occasionally in some runs, which are not included in the present analysis.

In other computational studies of single base flips from C:G and T:A pairs similar to the one presented here, varying results are reported. Depending on the surrounding sequence, the force field, and opening restraint applied the energetic cost for flipping thymine (from T:A) has been calculated to about 13 kcal, and 15–22 kcal have been computed for flipping cytosine (in C:G) [10], [11], [28]. Despite the variation of the detailed numbers in all the computational studies, the single base flip of mispaired T requires less energy than base flip of a pyrimidine from T:A or C:G Watson-Crick pairs. This is in agreement with the order of equilibrium constants for base pair opening, obtained from experimental imino proton exchange rates [65], which are larger by two orders of magnitude in T:G than in T:A and by one order of magnitude in T:A than in C:G.

However, as has been pointed out previously [10]–[12], imino proton exchange can take place already at an opening angle of approximately 30° from equilibrium (i.e. 70° flip angle). Our results are in agreement with this finding, showing that at a flip angle of 70° both, solvent accessibility of the imino proton (N3-H) and the number of its hydrogen bonds to water, increase significantly. In case of the mispaired T, the conformation with a 70° flip angle is populated even without the application of an external force allowing hydrogen bonds between the imino proton and solvent water to be observed. This would explain the unusually long life time of the “open state” (as determined by proton exchange kinetics) reported in [12]: The partially-open state with 70° flip angle is a second, metastable state of the T:G wobble pair. In the Watson-Crick pairs, 70° flip angle conformations are not stable and are about 6 kcal/mol higher in energy and as such unlikely to be observed. The partially open/flipped-out state (70° flip angle) clearly shows how the dynamics of the DNA is changed due to the G:T mispair.

One can speculate that the intrinsic dynamics of mispaired DNA plays a role in discriminating by the enzyme. The partially-open state, observed in our simulations, would serve as an indicator of mispared T in G:T, as opposed to A:T which could be recognised by the repair enzyme in a more passive mechanism: The Glycosylases which process T:G mispairs first recognise local distortions in the base steps and base-pair geometries which deviate from normal B-form DNA. Moreover, a partially open state of the T:G mispair, which we observe to be transiently occupied also in the unbiased simulations, is supposedly easy to be recognized by the searching repair enzyme.

Recognition of the helical distortions as exhibited by the mispaired T:G, as opposed to T:A, and subsequent formation of a tight “interrogative” [5] protein-T:G complex, may help to save the enzyme from processively trying to flip each base and thereby also avoid flipping of a paired (T:A) thymine and to erroneously remove it.

### Conclusion

DNA containing a single T:G mispair exhibits local dynamics significantly different from DNA without such a mispair. The T:G wobble pair shows a distorted conformation compared to T:A or C:G pairs. Moreover, besides the completely intra-helical state, it exhibits a second, less probable metastable state which is partially open/flipped-out and allows the thymine imino proton to be accessed by the solvent water.

Our free energy calculations show that thymine is much more probable to be flipped than cytosine in a C:G pair or thymine in a T:A pair, a fact that could possibly be exploited by the repair enzymes.

## Supporting Information

Figure S1 RMSD of the DNA with T:G mispair (red) and C:G (green) and T:A (black) pair, respectively, as a function of the simulation time.
(EPS)

Figure S2 Free energy profiles of local base step parameters of the T:G mispair (red) and C:G (green) and T:A (black) pair, respectively, as a function of the simulation time: a) roll b) tilt, and c) twist, d) rise, e) shift, f) slide.
(EPS)

Figure S3 Free energy profiles of local base pair parameters of the T:G mispair (red) and C:G (green) and T:A (black) pair, respectively: a) buckle b) opening, c) propeller, d) shear, e) stretch, and f) stagger.
(EPS)

Figure S4Free energy profiles of the 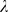-angles computed from the unbiased MD simulations. a) 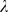-angle at T or C, respectively, b) 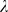-angle at A or G of the complementary strand.(EPS)

Figure S5 Free energy profiles of the twist angle of the T:G mispair (red, a)–e)) and T:A (black, f)–k)) and C:G (green, l)–q)) pair, respectively, at different simulation times. a,f,l) step 12, b,g,m) step11, c,i,o) step 10, d,j,p) step 9 and e,k,q) step 8.
(EPS)

Figure S6 Free energy profiles of the tilt angle of the T:G mispair (red, a)–e)) and T:A (black, f)–k)) and C:G (green, l)–q)) pair, respectively, at different simulation times. a,f,l) step 12, b,g,m) step11, c,i,o) step 10, d,j,p) step 9 and e,k,q) step 8.
(EPS)

Figure S7 Free energy profiles of the roll angle of the T:G mispair (red, a)–e)) and T:A (black, f)–k)) and C:G (green, l)–q)) pair, respectively, at different simulation times. a,f,l) step 12, b,g,m) step11, c,i,o) step 10, d,j,p) step 9 and e,k,q) step 8.
(EPS)

Figure S8 Free energy profiles of rise of the T:G mispair (red, a)–e)) and T:A (black, f)–k)) and C:G (green, l)–q)) pair, respectively, at different simulation times. a,f,l) step 12, b,g,m) step11, c,i,o) step 10, d,j,p) step 9 and e,k,q) step 8.
(EPS)

Figure S9 Free energy profiles of slide of the T:G mispair (red, a)–e)) and T:A (black, f)–k)) and C:G (green, l)–q)) pair, respectively, at different simulation times. a,f,l) step 12, b,g,m) step11, c,i,o) step 10, d,j,p) step 9 and e,k,q) step 8.
(EPS)

Figure S10 Free energy profiles of shift of the T:G mispair (red, a)–e)) and T:A (black, f)–k)) and C:G (green, l)–q)) pair, respectively, at different simulation times. a,f,l) step 12, b,g,m) step11, c,i,o) step 10, d,j,p) step 9 and e,k,q) step 8.
(EPS)

Figure S11 Free energy profiles of opening of the T:G mispair (red, a)–e)) and T:A (black, f)–k)) and C:G (green, l)–q)) pair, respectively, at different simulation times. a,f,l) base pair 12, b,g,m) base pair 11, c,i,o) base pair 10, d,j,p) base pair 9 and e,k,q) base pair 8.
(EPS)

Figure S12 Free energy profiles of propeller twist of the T:G mispair (red, a)–e)) and T:A (black, f)–k)) and C:G (green, l)–q)) pair, respectively, at different simulation times. a,f,l) base pair 12, b,g,m) base pair 11, c,i,o) base pair 10, d,j,p) base pair 9 and e,k,q) base pair 8.
(EPS)

Figure S13 Free energy profiles of buckle of the T:G mispair (red, a)–e)) and T:A (black, f)–k)) and C:G (green, l)–q)) pair, respectively, at different simulation times. a,f,l) base pair 12, b,g,m) base pair 11, c,i,o) base pair 10, d,j,p) base pair 9 and e,k,q) base pair 8.
(EPS)

Figure S14 Free energy profiles of stagger of the T:G mispair (red, a)–e)) and T:A (black, f)–k)) and C:G (green, l)–q)) pair, respectively, at different simulation times. a,f,l) base pair 12, b,g,m) base pair 11, c,i,o) base pair 10, d,j,p) base pair 9 and e,k,q) base pair 8.
(EPS)

Figure S15 Free energy profiles of shear of the T:G mispair (red, a)–e)) and T:A (black, f)–k)) and C:G (green, l)–q)) pair, respectively, at different simulation times. a,f,l) base pair 12, b,g,m) base pair 11, c,i,o) base pair 10, d,j,p) base pair 9 and e,k,q) base pair 8.
(EPS)

Figure S16 Free energy profiles of stretch of the T:G mispair (red, a)–e)) and T:A (black, f)–k)) and C:G (green, l)–q)) pair, respectively, at different simulation times. a,f,l) base pair 12, b,g,m) base pair 11, c,i,o) base pair 10, d,j,p) base pair 9 and e,k,q) base pair 8.
(EPS)

Figure S17 Free energy profiles of the flipe angle of a) the T:G mispair (red), b) T:A (black) and c) C:G (green) pair, respectively, at different simulation times.
(EPS)

Figure S18 Time series of the flip angle in T:G, computed from the unbiased MD simulations.
(EPS)

Figure S19 PMF of the Flip Angle at different simulation times.
(EPS)

Figure S20 Free energy profile of individual ABF simulations of the base flip in T:G.
(EPS)

Figure S21 Snapshots of the DNA base flip simulation of C:G (top), T:G (middle), and T:A (bottom), taken at (left) the flipped-in state (free energy minimum), (middle) at 60–70° flip angle and (right) at the flipped-out state.
(EPS)
